# The notion of the numeral base in linguistics

**DOI:** 10.1098/rstb.2024.0208

**Published:** 2025-10-20

**Authors:** Russell Barlow

**Affiliations:** ^1^ Department of Linguistic and Cultural Evolution, Max Planck Institute for Evolutionary Anthropology, 04103 Leipzig, Germany

**Keywords:** numerals, base, anchor, quinary, decimal, linguistic numeral systems

## Abstract

This article details how the notion of the numeral base has been used by linguists, both in the description of individual languages and as a broader concept for comparing different linguistic numeral systems. Although common linguistic understandings of ‘base’ are generally not the same as mathematical definitions of the term, they may help us understand the different ways in which languages encode numbers. In the hopes of supporting efforts in comparative linguistics as well as facilitating interdisciplinary studies of numeral systems, this article presents a typology of the various phenomena that may all be considered to be a ‘base’.

This article is part of the theme issue ‘A solid base for scaling up: the structure of numeration systems’.

## 1. Introduction

Linguists are interested in systematic patterns in the structure of languages, including the ways in which different elements may be combined to create meaning. Thus, among other things, linguists examine the various ways in which words are arranged into sentences in different languages and the different ways in which words are themselves composed of smaller linguistic elements. Languages often employ conventionalized means of referring to exact quantities, and these linguistic expressions—called numerals—may exhibit systematic patterns whereby elements are combined to create numerical meaning. As such, numeral systems are also of interest to linguists, who have long used the term base (along with related terms like decimal, quinary and vigesimal) to describe certain structural patterns found in these linguistic systems. However, the use of these terms in the linguistic literature does not always match their use in other fields. Moreover, these terms are often used differently in the typological linguistic literature, which is concerned with comparing features across multiple languages, from how they are used in the descriptive linguistic literature, which is concerned with describing individual languages.

This article aims to clarify the different yet related features that linguists have in mind when employing terms like base, both when describing individual languages and when conducting typological studies comparing numeral systems across multiple languages. I organize the various features that may be considered a numeral base in linguistic systems into a proposed typology, hopefully improving conceptual clarity both within linguistics and across disciplines.

## 2. The use of ‘base’ in descriptive linguistic literature

Descriptions of individual languages are written by various kinds of authors: professional linguists, PhD students, pedagogues, missionaries, government employees and other individuals who are not necessarily adhering to a terminological standard. Thus, while terms like base, quinary and decimal are commonly found in the descriptions of linguistic numeral systems, they are not necessarily understood in the same way by all who use these terms. Unfortunately, such terms are rarely defined in grammatical descriptions, allowing us only to infer what was meant by a given author. In the best cases, a grammatical description contains a long list of numerals in the language: perhaps the words for ‘1’ through ‘100’, as well as ‘1000’ and even some higher numbers. Other times, however, we are only given a few numerals and a statement like ‘Language X has a quinary numeral system’. What does this mean?

Although rarely defined in the descriptive linguistic literature, terms like quinary (or base-5) and decimal (or base-10) often reflect a shared understanding by linguists of what is important in the study of linguistic strategies of referring to exact quantities. In short, linguists have frequently used the term base in a broader sense than is used in mathematics and other fields (for the mathematical understanding of base, see [1]). Perhaps the broadest general linguistic understanding of base can be summarized as follows:

A base is a numerical value that is used systematically in the formation of numerals.

The term numeral is to be understood here in a linguistic context: a numeral is a word that refers to an exact quantity [2]. This description specifies the systematic use of the base for two reasons. First, language users may have various means of referring to quantities, including ad hoc or idiosyncratic formulations. Therefore, to be considered to be part of ‘the language’, these numerical expressions must be conventionalized.^1^ Second, in order to be considered a base, the numerical value in question should be used with some degree of systematicity (or regularity) within the system. For example, a linguist would probably not describe a system as ‘base-5’ if the number 5 is used in the formation of only one numeral other than 5.^2^ At the same time, however, complete and utter regularity is rare within the grammatical domains of natural language, so most linguists would probably accept a system as ‘base-5’ even if there is one exception to the ‘base’-defining rules.^3^ Thus, terms like systematic and regular should be understood as scalar concepts: numeral systems may be more or less systematic or regular. The degree of systematicity or regularity required for identifying a base may vary according to author or description.

Several linguists have acknowledged the incongruence between the mathematical notion of the ‘base’ and many linguists’ tacit understanding of the term in a linguistic context. For example, Lynch [7] writes:

Lincoln [8] notes that so-called quinary systems are not really quinary or base-5 in the mathematical sense, since the ‘milestones’ are not 5, 5² = 25, 5³ = 125, etc. However, this term has been so widely used in Oceanic studies that I will retain it here.

Although terms like base-5 and quinary are commonly used in describing linguistic numeral systems, it seems to be the case—by the mathematical definition of base—that no natural human language makes regular and exclusive use of a conventionalized base-5 system. Putative cases of base-5 systems generally instead reflect what might be considered sub-bases of 5 (e.g. a base-10 system with a sub-base of 5 or a base-20 system with a sub-base of 5).^4^

Similarly, linguists sometimes use the term binary to refer to systems that use the number 2 as an element for constructing higher numerals despite not using the higher powers of 2 (e.g. 2² = 4) in a similar way. In New Guinea, for example, where such systems are relatively common, it is unusual for people to use these ‘binary’ means for counting higher than 4 or 5. Often, there are no conventionalized terms for such higher numbers. Sometimes reference is made to a word meaning ‘hand’ for numbers greater than or equal to 5. For example, Holzknecht [10] refers to the Markham languages (Austronesian family, Papua New Guinea) as employing ‘binary number systems’, to which Ross [11] notes:

However, it is not strictly correct to call this system ‘binary’, as a binary system requires that a new base intervenes at 4. The concept of a ‘base’ requires that the next higher base (or the highest conventional numeral) be a multiple of the lower base, and 5, the next higher base, is not a multiple of 2 (but 4 would be). Thus 2 is not a base, but simply an element from which 3 and 4 are built in each quinary round [12].

## 3. The use of ‘base’ in typological linguistic literature

Although linguists describing individual languages do not always define their terms, typological linguists, whose job is to compare linguistic features across multiple languages, must work with clearly defined terms that can be applied crosslinguistically.

In the typological literature, a commonly cited definition for base is that of Comrie [13]: ‘the value *n* such that numeral expressions are constructed according to the pattern . . . *xn + y*, i.e. some numeral *x* multiplied by the base plus some other numeral’.^5^ Hanke [15], citing Comrie [13], refers to this sort of numerical expression as an additive-multiplicative base. Other mentions of this definition are commonly found [16–19].

Comrie’s definition thus requires the presence of both addition and multiplication. In an earlier definition, Greenberg [20] appeals only to multiplication, defining the base as a ‘serialized multiplicand’, which, in turn, he defines as ‘a number whose successive multiplication by at least two other numbers results in serialized products which are either expressed as simple lexemes or as a product of the multiplicand and multiplier, and such that each serialized product is also a serialized augend or minuend’.

Another definition of base comes from Hammarström [5,12]:^6^

The number *n* is a base iff

(1) the next higher base (or the end of the normed expressions) is a multiple of *n*; and

(2) a proper majority of the expressions for numbers between *n* and the next higher base are formed by (a single) addition or subtraction of *n* or a multiple of *n* with expressions for numbers smaller than *n*.

These definitions are more restrictive than some commonly used interpretations of base in the descriptive linguistic literature. Whereas some linguistic descriptions apply the term base to cases where the number in question is only used in additive operations, these definitions require a base either to be used (also) in multiplicative operations or to have special designations occurring at one or more multiples of itself. This is reminiscent of mathematical definitions of base, in which it is required that one or more powers of the base be specially defined [22]. In linguistic terms, we would thus expect a base-5 system to have a word for 5² (i.e. 25) that is not derived from any other numerals, similarly to how in base-10 systems—such as the one used in English—there is an underived word for 10² (i.e. 100; e.g. *hundred*). However, such mathematically defined base-5 systems are essentially unattested among natural languages. Thus, commonly described ‘quinary’ systems—provided they have conventionalized expressions for sufficiently large numbers—tend to switch to using 10 or 20 (or both) as elements for building numerals with values higher than 10 or 20.^7^

On the other hand, the descriptive uses of terms like binary, quinary, decimal and so on (as discussed in §2) tend to be more in keeping with the notion of cycles, as put forward by Salzmann [23]:

The *cyclic* pattern is a succession of morphemes or groups of morphemes according to which the numerical system is analyzable in terms of one or more similar or regular sets of recurring morphemes or groups of morphemes. This pattern covers all systems that are referred to as binary, ternary, … decimal, etc. By no means do we intend to abandon the use of these terms; however, it is suggested that they be used in structural analysis with care, since it often happens that a cycle does not function consistently thruout a system, being either modified or changed. Also, as has been shown above, the comparative value of a cycle is considerably limited.

There have been several references to Salzmann’s [23] use of cycle in the linguistic literature [11,19,20,24,25]. A very early definition of base that seems kindred to this notion of cycle is that of Conant [26]: ‘… a number from which [one] makes a fresh start, and to which [one] refers the next steps in [one’s] count …’. Similarly, Stampe [27] writes: ‘The *base* number of a number system, then, must be defined as that number from which counting starts over. In the vast majority of languages, at least originally, it is the highest of the simple numbers.’

Plank [16] makes a helpful distinction between what he calls a construction-base and what he calls a cycle-base:

Numerals are frequently referred to as ‘bases’ when they are an atomic expression (or at any rate not transparently compositional, synchronically speaking) and when expressions for other numerals are formally based on them, with higher or lower numerals constructed by arithmetic operations (addition, subtraction, multiplication) with their help. The requirement of such a construction-base being itself atomic is sometimes waived … A cycle-base is the narrower concept: a numeral is a cycle-base if it is a construction-base and cyclically recurs in linguistic designations of multiples of the respective number … and/or in exponentiation with that base …

Finally, we may mention Hurford’s [28,29] work, since it has been influential in crosslinguistic accounts of numeral systems. Although he uses the term base, Hurford [29] is generally concerned with the formal composition of individual complex numerals; thus, he provides a pragmatic suggestion for identifying a base in a given numeral: ‘Let us label the highest-valued word in a numeral construction the “base-word”.’

## 4. A typology of ‘bases’

Plank’s [16] terminological distinction does an excellent job of distinguishing the two broad uses of the term base in the linguistic literature. However, each term is capable of being more or less strictly defined. In this section, I further define and typologize these two broad categories, showing how they can be related to Pelland’s [30] term anchor, which is a broader category that includes not only bases but also other numerical values that are used in structuring numeral systems. The definitions I provide below represent a narrower interpretation of ‘anchor’, meant to apply to linguistic numeral systems in particular. First, I would define construction base as follows:

### (a) Construction base (or anchor)

A construction base (or anchor) is a numerical value to which an arithmetic operation is applied so as to form a numeral.^8^ Table 1 provides abstract examples of systems in which different numbers serve as anchors.

**Table 1.** Examples of anchors. Complex numerals involving the specified anchor are in bold.

anchor	numerals	example of anchor
4	{1, 2, 3, **1·4**, **4+1**, **4+2**, **4+3**, **2·4**, …}	**2·4** = 8
5	{1, 2, 3, 4, 5, **5+1**, 7, **5+3**, 9, …}	**5+3** = 8
6	{1, 2, 3, 4, 5, 6, 7, **2+6**, **3+6**, **4+6**, …}	**2+6** = 8
10	{1, 2, 3, 4, 5, 6, **10−3**, **10−2**, **10−1**, …}	**10−2** = 8

It may be helpful to identify and define several subcategories of construction base (or anchor), such as the following.

### (b) Regular construction base (or regular anchor)

A regular anchor is a numerical value *n* that functions as the anchor of at least half the numerals other than *n* within a cycle.^9^ For an additive anchor, a cycle could be defined as the set of numerals greater than (*n*)·(*a*) and less than or equal to (*n*)·(*a*+1), where *a* is a positive integer. For example, the number 10 (= *n*) meets the requirements of a regular additive anchor if it is employed as the additive anchor in at least five (= *n*÷2) numerals greater than 10 (= (*n*)·(1)) and less than or equal to 20 (= (*n*)·(1+1)).^10^ The number 10 (= *n*) would also be considered a regular additive anchor if it occurred, for example, in five (= *n*÷2) numerals greater than 70 (= (*n*)·(7)) and less than or equal to 80 (= (*n*)·(7+1)), provided that the operation of addition in those numerals is applied to a numeral derived from 10 (e.g. 72 = (7·10)+2). In practice, we may be mostly interested in the range from *n*+1 to 2*n*.

For a subtractive anchor, a cycle could be defined as the set of numerals greater than 0 and less than *n* or the set of numerals greater than or equal to (*n*)·(*a*) and less than (*n*)·(*a*+1), where *a* is a positive integer. For example, the number 5 (= *n*) meets the requirements of a regular subtractive anchor if it is employed as the subtractive anchor in at least three (≥ *n*÷2) numerals between 0 and 5 (= *n*).^11^ As another example, 10 (= *n*) meets the requirements of a regular subtractive anchor if it is employed as the subtractive anchor in at least five (= *n*÷2) numerals greater than 10 (= (*n*)·(1)) and less than or equal to 20 (= (*n*)·(1+1)), provided that the operation of subtraction in those numerals is applied to a numeral derived from 10 (e.g. 17 = (2·10)−3). Regular subtractive anchors are probably very uncommon in numeral systems.

For a multiplicative anchor, a cycle could be defined as the set of multiples of *n* between *nᵃ* and *n*⁽*^a^*^+1^⁾, inclusive, where *a* is a positive integer. For example, the number 10 (= *n*) meets the requirements of a regular multiplicative anchor if it is employed as the multiplicative anchor in at least five (= *n*÷2) multiples of 10 (= *n*) between 10 (= *n*¹) and 100 (= *n*⁽¹^+^¹⁾).^12^ For this definition, we may wish to accept systems in which multiplication by 1 is not overtly expressed (e.g. systems in which *n* is expressed as ‘10’, as well as systems in which *n* is expressed as ‘1·10’).

Although theoretically possible, languages are not known to employ divisive anchors (there are, however, a few attestations of derivation by means of multiplication by a fraction, as in ‘100·½’ for ‘50’). It is unclear how the cycle for a regular divisive anchor should be defined. Likewise, it is unclear what, if anything, should be considered a ‘cycle’ for an exponential anchor (or base). Table 2 provides some additional abstract examples of regular anchors using different operations.

**Table 2.** Examples of regular anchors. The relevant cycle in each case is underlined. Complex numerals involving the specified anchor are in bold.

anchor	numerals	occurrences within a cycle
2	{1, 2, 3, **2+2**, 5, …}	2 is an additive anchor in one numeral (= ¹⁄₂ of numerals in cycle)
3	{1, 2, 3, **3+1**, **3+2**, 5+1, 5+2, …}	3 is an additive anchor in two numerals (= ²⁄₃ of numerals in cycle)
4	{1, 2, 3, **[1⋅]4**, …, **2⋅4**, …, **3⋅4**, …, 16, …}	4 is a multiplicative anchor in two (or three?) numerals (= ²⁄₄ or ³⁄₄ of numerals in cycle)
5	{1, **5−3**, **5−2**, **5−1**, 5, 5+1, 5+2, 5+3, 5+4, …}	5 is a subtractive anchor in three numerals (= ³⁄₄ of numerals in cycle)

### (c) Canonical construction base (or canonical anchor)

A canonical anchor is a numerical value *n* that functions as the anchor of all numerals in a cycle aside from the *start* and *end* values of the cycle. For example, 5 can be considered a canonical additive anchor if it functions as the additive anchor in all of {6, 7, 8, 9}. As another example, 5 would be considered the canonical additive anchor if it occurs as the additive anchor in all of {11, 12, 13, 14}, provided that the operation of addition in those numerals is applied to a numeral derived from 5 (e.g. 12 = (2·5)+2). It is not required for the ‘end values’ (e.g. *n*, 2*n*) to be formed with *n* as an additive anchor. Thus, 5 would be considered a ‘canonical’ additive anchor in all three of the following sets of numerals:

— {1, 2, 3, 4, 5, 5+1, 5+2, 5+3, 5+4, 10}
— {1, 2, 3, 4, 5, 5+1, 5+2, 5+3, 5+4, 2·5}
— {1, 2, 3, 4, 5, 5+1, 5+2, 5+3, 5+4, 5+5}

As another example, 10 can be considered a canonical multiplicative anchor if it functions as the multiplicative anchor in all of {20, 30, 40, 50, 60, 70, 80, 90}. It may, but need not, also occur as a multiplicative anchor in the ‘end values’ of the cycle, as, for example, ‘10·1’ for ‘10’ and ‘10·10’ for ‘100’.

Perhaps the most ‘canonical’ type of numeral system is one in which the same value functions as a canonical additive anchor in the system’s additive cycles and as a canonical multiplicative anchor in the system’s multiplicative cycles.^13^ A canonical anchor is a particular type of regular anchor. Table 3 provides additional abstract examples of canonical anchors using either addition or multiplication.

**Table 3.** Examples of canonical anchors. Complex numerals involving the specified anchor are in bold.

anchor	numerals
2 (additive)	{1, 2, **2+1**, **2+2**, …}
3 (additive)	{1, 2, 3, **3+1**, **3+2**, 2·3, …}
4 (multiplicative)	{1, 2, 3, 4, … **2·4**, … **3·4**, … 16, …}
5 (multiplicative)	{1, 2, 3, 4, **5·1**, … **5·2**, … **5·3**, … **5·4**, … **5·5**, …}

### (d) Sporadic construction base (or sporadic anchor)

A sporadic anchor is a numerical value *n* that functions as the anchor of less than half the numerals other than *n* within a cycle. All anchors that are not regular are sporadic. Table 4 provides abstract examples of sporadic additive anchors.

**Table 4.** Examples of sporadic anchors. The relevant cycle in each case is underlined. Complex numerals involving the specified anchor are in bold. Note that each example actually contains multiple anchors.

anchor	numerals	occurrences within a cycle
4	{1, 2, 3, 4, 5, 5+1, **4+3**, 5+3, 5+4, 5+5, …}	4 is an anchor in one numeral (= ¹⁄₄ of numerals in cycle)
5	{1, 2, 3, 4, 5, 6, 7, **3+5**, **4+5**, 10, 10+1, …}	5 is an anchor in two numerals (= ²⁄₅ of numerals in cycle)
6	{1, 2, 3, 4, 5, 6, **6+1**, **6+2**, 5+4, 10, 10+1, 10+2, 10+3, …}	6 is an anchor in two numerals (= ²⁄₆ of numerals in cycle)

### (e) Hapax construction base (or hapax anchor)

A hapax anchor is a numerical value *n* that functions as the anchor in exactly one numeral other than *n* within a cycle. A hapax anchor is (usually) a particular type of sporadic anchor.^14^ Table 5 provides abstract examples of hapax additive anchors.

**Table 5.** Examples of hapax anchors. Complex numerals involving the specified anchor are in bold.

anchor	numerals
3	{1, 2, 3, **3+1**, 5, 5+1, 5+2, …}
4	{1, 2, 3, 4, 5, 5+1, **4+3**, 5+3, 5+4, 5+5, …}
5	{1, 2, 3, 4, 5, 6, **5+2**, 8, 9, 10, 10+1, 10+2, …}

Thus, all anchors (or construction bases) are either regular or sporadic. Canonical anchors are a subtype of regular anchors. Hapax anchors are a subtype of sporadic anchors. (However, exceptionally, the anchor value 2 may be both hapax and regular.)

Terms like augend, minuend, multiplicand and dividend can be expressed in terms of various types of construction bases or anchors, as shown in table 6.

**Table 6.** Anchors described by their arithmetic operation.

anchor	operand
additive anchor	augend
subtractive anchor	minuend
multiplicative anchor	multiplicand
divisive anchor	dividend
exponential anchor	[exponential] base

Continuing with Plank’s [16] terminological distinction, a definition for cycle base may be formulated as follows:

### (f) Cycle base

A cycle base is a regular construction base to which an arithmetic operation is applied so as to form another regular construction base. The cycle base is thus closer to the mathematical notion of a base. In practice, in linguistic numeral systems, the arithmetic operation used in regular construction bases is almost always—if not always—addition; and the (secondary) arithmetic operation used in forming one or more other regular construction bases is almost always—if not always—multiplication. Thus, the notion of cycle base is practically synonymous with the notion of additive-multiplicative base [15]. However, a cycle base could, in theory, represent an additive-additive base (in other words, an additive cycle base). Subcategories of cycle bases could include those shown in table 7.

**Table 7.** Theoretical subcategories of cycle bases, each exemplified with ‘5’.

type of cycle base	example with 5 as the base
additive cycle base	{1, 2, 3, 4, 5, 5+1, 5+2, 5+3, 5+4, 5+5, 5+5+1, 5+5+2, 5+5+3, 5+5+4, 5+5+5, …}
multiplicative cycle base	{1, 2, 3, 4, 5, 5+1, 5+2, 5+3, 5+4, 2·5, (2·5)+1, (2·5)+2, (2·5)+3, (2·5)+4, 3·5, …}
exponential cycle base	{1, 2, 3, 4, 5, 5+1, … 2·5, … 3·5, … 4·5, … 5², 5²+1, …}

I question the degree to which additive cycle bases are used in conventionalized systems. Although recorded (to some degree) for some languages with a construction base of 5 (e.g. Southwest Tanna, Austronesian family, Vanuatu), these formulations might be ad hoc.^15^

The multiplicative cycle base is the ‘canonical’ cycle base. Linguistic numeral systems that employ a multiplicative cycle base are the systems most widely accepted—both by linguists and by researchers in other disciplines—as representing ‘true’ base systems.

Unlike the operations of addition and multiplication, exponentiation is perhaps never overtly expressed in linguistic numeral systems. Thus, although a word meaning ‘17’ may be composed of morphemes meaning ‘10’ and ‘7’ (addition), and a word meaning ‘30’ may be composed of morphemes meaning ‘3’ and ‘10’ (multiplication), I know of no language in which a word meaning ‘100’ is composed of morphemes meaning ‘10’ and ‘2’. When languages do employ special designations for powers of a base, this is generally done by means of an atomic term, underived from any other numeral, as in English *hundred* and *thousand*, rather than something like *ten-to-the-two* and *ten-to-the-three*.^16^

Consider, for example, Mandarin, in which the arguments of the operations of addition and multiplication (although not the operators themselves) are transparently stated in complex numerals. In the Mandarin numeral *shí qī* ‘17’ (literally ‘10[+]7’), the additive construction base is the element *shí* ‘10’. In the Mandarin numeral *sān shí* ‘30’ (literally ‘3[·]10’), the multiplicative cycle base is (also) the element *shí* ‘10’. And in the Mandarin numeral *sān shí qī* ‘37’ (literally ‘(3[·]10)[+]7’), the element *shí* ‘10’ serves as a base simultaneously for addition and for multiplication. However, 10² is expressed as bǎi ‘hundred’, which is an opaque term—that is, it contains neither the exponential cycle base *shí* ‘10’ nor the exponent *èr* ‘2’.^17^

## 5. Conclusion

Why do linguists call things bases that are not mathematical bases? The terminology may be unfortunate, but the reason suits the goals of the field. Numerical elements are combined in languages in interesting ways, often irrespective of any mathematical base. Indeed, many linguistic numeral systems lack bases entirely (in the sense used in other fields) and yet contain structured patterns. Many of these structures recur across the world’s languages, such as using 2 or 5 as elements for constructing higher numerals, whereas other logically possible structures are all but unattested—for example, using 3 or 7 in similar ways. To investigate only mathematical bases would overlook many of the recurring patterns found in numerical expressions in the world’s languages.

## Data Availability

This article has no additional data.

## Footnotes

^1^Note, however, that linguistic descriptions of ‘numeral systems’ are often written with little consideration of conventionalization. Sometimes the only information available on the numerals in a given language is derived from a single elicitation session in which a linguist, anthropologist or missionary has interviewed a single speaker. It is thus not always possible to know the extent to which any putative system is conventionalized within a language. On conventionalization in numeral systems, see von Mengden [3]. For the issues of disentangling conventionalization in language from other forms of representation, see the appendix. A reviewer notes that, in addition to conventionalization, it may be useful to consider the parameter of whether a linguistic expression is basic or non-basic (no relation to the term base): a basic linguistic expression is monomorphemic, conveys the primary meaning of its form and is not greatly restricted in usage. Like non-basic colour terms, non-basic numerical expressions can contain multiple morphemes (e.g. *forest green*, *twenty-three*), represent an extension of meaning (e.g. *peach* for the colour of the fruit, *k* for the quantity associated with the prefix *kilo-*) or be highly contextually restricted (e.g. *blond* for hair colour, *dozen* for standardized sets of certain things like eggs and roses).

^2^E.g.: {1, 2, 3, 4, 5, 6, 7, **5+3**, 9, 10, 10+1, 10+2, 10+3, …}. Occasionally, however, the term base is indeed used for cases such as this. See §4 for terminological suggestions. Also, note that—for purposes of brevity and clarity—this article largely relies on abstract examples of numeral systems (such as the one in this endnote) rather than data from actual languages. In these examples, an arithmetic operation suggests that a particular numeral is derived from that operation, whether explicitly or implicitly. Thus, for example, if instead o*f eight*, English had a numeral of the form ‘5+3’, it might have a form such as one of the following*: five-plus-three*, *five-and-three*, *fivethree* (with the operation implied), *fithre* (with phonological changes), *plus-three* (with the number 5 implied), *handthree* (where the number 5 is represented by a form other than *five*), etc. For a novel alternative way of abstractly representing numeral systems, see Schlimm [4]. For attested examples of linguistic numeral systems with different types of ‘base’ configurations, see Hammarström [5] and Comrie [6].

^3^E.g.: {1, 2, 3, 4, 5, 5+1, 5+2, **8**, 5+4, 2⋅5, (2⋅5)+1, (2⋅5)+2, (2⋅5)+3, …}.

^4^A sub-base may be considered a type of anchor (see §4 and Chrisomalis [9]).

^5^Comrie [14] has also offered the following definition of base: ‘that numerical value to which various arithmetic operations are applied’. If we understand ‘various’ to mean ‘multiple, different’ (e.g. both addition and multiplication are applied to the numerical value), then this definition is rather similar. However, ‘various’ seems to have been interpreted by some as meaning ‘any of several’, suggesting a much looser definition (i.e. a value to which an operation like addition or multiplication is applied).

^6^Cf. Schapper & Hammarström’s [21] definition of base-5 systems as those in which more than half (i.e. ≥ 3) of the expressions for 6 through 9 are formed with 5.

^7^Thus, in such a ‘quinary’ system, the number 25 is more likely expressed as something like (2⋅10)+5 or ([1⋅]20)+5. There have been a few mentions of numeral systems in which 25 is expressed as 5⋅5, but even these are somewhat questionable, perhaps being idiosyncratic or novel utterances [5]. Even still, I know of no language with a putative base-5 system attested as having a term for 25 that would be analogous to a base-10 system’s treatment of 100 (e.g. as *hundred*, as opposed to *ten-ten*).

^8^
This ‘arithmetic operation’ need not be explicitly represented—in other words, there is no requirement for numerals to contain an overt morpheme corresponding to something like a plus sign or multiplication sign. Furthermore, no arithmetic abilities are required of the language user—that is, a person can use numerals formed with anchors without consciously performing any mathematical operations. The ‘arithmetic operation’ exists insofar as the analyst of the numeral system is able to describe it. An additional question may be raised: when there are two numerical values represented in an expression, how do we know which is the value ‘to which’ the arithmetic operation is applied? For example, why would we claim that 4, rather than 2, is the anchor in 2⋅4? Often the decision can be made according to system-internal evidence. Imagine, for example, that this 2⋅4 construction is part of the following system: {1, 2, 3, 1⋅4, 4+1, 4+2, 4+3, 2⋅4, (2⋅4)+1, (2⋅4)+2, (2⋅4)+3, 3⋅4, …}. In this case, we would have two clues that 4 is an anchor. First, the operation of multiplication occurs not simply one time (in 2⋅4) but repeatedly (for all multiples of 4). Although 2 is used in multiplication, it is done so only once in this series, as are 1 and 3. Second, 4 functions as an anchor not only when multiplication occurs but also when addition occurs, again regularly. Furthermore, a possible heuristic may be that whenever exactly two numerals occur in a complex numeral expression, the larger is probably the anchor. Finally, not every combination of multiple numerals in a complex numeral expression necessarily contains an anchor. So-called pairing methods do not seem to be profitably analysable as containing an anchor. Consider, for example, the following system: {1, 2, 3, 4, 5, 3+3, 1+3+3, 4+4, 4+4+1, 5+5, …}. In this system, 6, 8 and 10 are formed by pairing (or doubling) identical numeral elements. There is no system-internal evidence for choosing as an anchor either of the two 3s in the numeral for 6. Perhaps they should both be considered anchors.

^9^
It should be noted that an anchor is a *number* not a *numeral*. It is possible for a *number* to be used to derive a complex numerical expression without the corresponding *numeral* occurring in the form of that complex numerical expression. For example, the number 10 is a multiplicative anchor in the English numeral *sixty*, even though the numeral *ten* does not occur in the expression (nor does it matter whether or not *-ty* is etymologically related to *ten*). See Pelland [30].

^10^
E.g.: {… 10, **10+1**, **10+2**, **10+3**, 15−1, 15, 15+1, **10+7**, **10+8**, (2⋅10)−1, 2⋅10, …}.

^11^
E.g.: {1, **5−3**, **5−2**, **5−1**, 5, 5+1, 5+2, 5+3, 5+4, …}.

^12^
E.g.: {… 10, **2⋅10**, **3⋅10**, **4⋅10**, 100÷2, (3⋅10)⋅2, ((3⋅10)⋅2)+10, **8⋅10**, **9⋅10**, 100, …}.

^13^
E.g. 4 in: {1, 2, 3, **4[⋅1]**, **4+1**, **4+2**, **4+3**, **4⋅2**, (**4⋅2)+1**, (**4⋅2)+2**, (**4⋅2)+3**, **4⋅3**, …}.

^14^
When the number 2 is an anchor, it is possible for it to be labelled both as ‘hapax’ and as ‘regular’ (as opposed to ‘sporadic’), since it could satisfy the regularity requirement by occurring in only one numeral other than the one representing 2. Take, for example, the following system: {1, 2, **2+1**, 4, 5, …}. Here, 2 is the anchor for only one numeral in the cycle, namely, 3.

^15^In Chan *et al.* [31], the following forms are given for Southwest Tanna: *kɨlkɨlɨp* ‘5’, *kɨlkɨlɨp kɨlkɨlɨp* ‘10’, *kɨlkɨlɨp kɨlkɨlɨp kɨlkɨlɨp* ‘15’ and *kɨlkɨlɨp kɨlkɨlɨp kɨlkɨlɨp kɨlkɨlɨp* ‘20’. Lynch’s [32] description of Southwest Tanna is, however, somewhat different: a ligature *mɨ* is said to be used after the form *kɨlkɨlɨp* in forming longer numerals and the word for ‘20’ is described as containing a word meaning ‘person’. In addition to possible examples of 5 being used as an additive cycle base, there are some examples of systems with a construction base of 2 being recorded as having additive cycle bases. For example, Orop (or Arop-Sissano, Austronesian family, Papua New Guinea) is recorded as encoding 10 as 2+2+2+2+2 [31]. Although formulations such as these (and occasionally even higher iterations of addition by 2) are recorded in wordlists, I highly doubt that speakers ever use them in natural discourse (or as part of a conventionalized system) (cf. [19]).

^16^
A reviewer points out that the long scale system can potentially be analysed as overtly expressing exponentiation (i.e. *mi-llion* = 1,000,000¹, *bi-llion* = 1,000,000², *tri-llion* = 1,000,000³, *quadri-llion* = 1,000,000⁴, etc.). This analysis is valid insofar as we can consider these Latinate prefixes to have numerical meaning in English or other languages that use the long scale system (*mi-* is not a Latinate prefix but comes from a reanalysis of *mille* ‘thousand’ in Italian *milione* ‘million’, literally ‘big thousand’).

^17^
In this Mandarin example, it seems difficult to construct 100 in such a way as to include *shí* ‘10’ and *èr* ‘2’ without introducing yet another morpheme. The string of morphemes *shí-èr* ‘10-2’ would be interpreted as 12 (i.e. ‘10+2’) and the string of morphemes *èr-shí* ‘2-10’ would be interpreted as 20 (i.e. ‘2⋅10’).

## Appendix. A note on linguistic versus non-linguistic systems

The focus of the discussion in this paper has been on linguistic numeral systems—in particular, on spoken (or signed) linguistic systems. Language can be conveyed using different representational formats (or ‘modalities’). Thus, language may be spoken, signed or written. The numerals encoded in written language can be covered by the same categories used in this article, provided they are genuinely linguistic systems (e.g. phonetic, phonemic or morphemic representations of a spoken or signed system) and not (only) notational systems (e.g. Western numerals^18^ carrying linguistic information only incidentally). The human body can also serve as a means of conveying language. Thus, signed numerals should be comparable with spoken numerals, since they are also part of a linguistic system. However, in addition to linguistic numeral systems, there are numeral systems that may be encoded in non-linguistic ways. For example, the body may be used to enumerate or signal exact quantities (e.g. via tallying or gesturing) in ways that do not fit within a larger linguistic system. Likewise, external materials, such as abacuses or tally sticks, may be used for enumerating. As far as I am aware, external (non-bodily) materials are never a part of linguistic numeral systems.^19^

 However, the boundaries between ‘linguistic’ and ‘non-linguistic’ systems are not always clear, especially since speech (or signing) can be used to refer to non-linguistic behaviour. Some cultures that do not employ conventionalized linguistic numeral systems for referring to higher numerals may nevertheless count via non-linguistic means, such as through digit-tallying. A speaker from such a culture could refer indirectly to an exact quantity through verbal reference to such a digit-tallying practice (e.g. ‘a hand and put two fingers atop it’ to mean ‘7’). However, such a linguistic expression would have to become conventionalized for it to be considered part of a linguistic *system* (e.g. if a speaker from this community could just as well refer to ‘7’ as ‘one full hand and two from the other’, then there is probably not a conventionalized expression for this number in this language). In practice, given such a possible historical pathway from tally-based physical practices to a linguistic system, this would probably entail some degree of lexicalization—that is, a more descriptive phrase would, over time, reduce in length and become fossilized as a single (perhaps multimorphemic) word.

 One interesting kind of body-based numeration method is found in the body-part tallying systems of New Guinea and Australia. In this method of counting, people point to different body parts to represent different numbers, generally starting by pointing to a finger of one hand, then proceeding through the other four fingers, then pointing to points along the arm, shoulder, neck and head, and then usually continuing symmetrically, pointing to the same places along the other side of the body. Insofar as the various points on the body have linguistic expressions associated with them, a member of a community that uses such a body-part tallying system could refer indirectly to a number by means of reference to a given body part (e.g. ‘elbow’ to mean ‘8’). However, only if such references become conventionalized lexical expressions should they be considered part of a linguistic system. Although there are a handful of descriptions of these body-part terms being used as integrated linguistic expressions [36,37], many descriptions of body-part tallying methods simply do not discuss whether or how the physical practice might relate to any linguistic system, and some descriptions explicitly state that body-part terms are not used for numerical references (e.g. by saying something like ‘I saw elbow pigs’ to mean ‘I saw eight pigs’). Laycock [38] notes: ‘There are no “numerals” in a tally system, so that one may not receive a reply to the question “how many?”, or find the points of the tally system qualifying nouns, as do true numerals.’ Hammarström [5] further points out that, where we have more detailed social information on communities that use body-part tallying systems, the systems are said to be used only in special ceremonial circumstances, and the tallying has to be conducted on a physically present body.

 Nevertheless, body-part tallying systems have been included in (otherwise purely linguistic) typological studies and databases. For example, ‘extended body-part systems’ are included in the *WALS* chapter on ‘numeral bases’ [13], where they are discussed as examples of linguistic numeral systems that lack arithmetic bases. Four languages are coded as having such systems. Similarly, Grambank [39] includes a feature ‘Is there a body-part tallying system?’ (GB336). In version 1.0, 44 languages are coded as having this feature. Although not treating body-part tallying as a linguistic system, Barlow [19] found 100 languages of mainland New Guinea and one language of the Torres Strait Islands for which there were indications of their speakers using body-part tallying systems. That study did not investigate speaker communities in mainland Australia, for which there is also evidence of body-part tallying systems—namely, within Kulin language communities (Pama-Nyungan family, Australia) [5].
